# Supplementary material for: Venomix: a simple bioinformatic pipeline for identifying and characterizing toxin gene candidates from transcriptomic data
Source: PeerJ. 2018 Jul 31;6:e5361. doi: 10.7717/peerj.5361 (PMC6074769; doi:10.7717/peerj.5361)
Supplement: Supplemental Information 6 [file peerj-06-5361-s006.gz › FinalOutput_1E-6/Disintegrin_lebein-1-beta_8/finaltree.pdf]

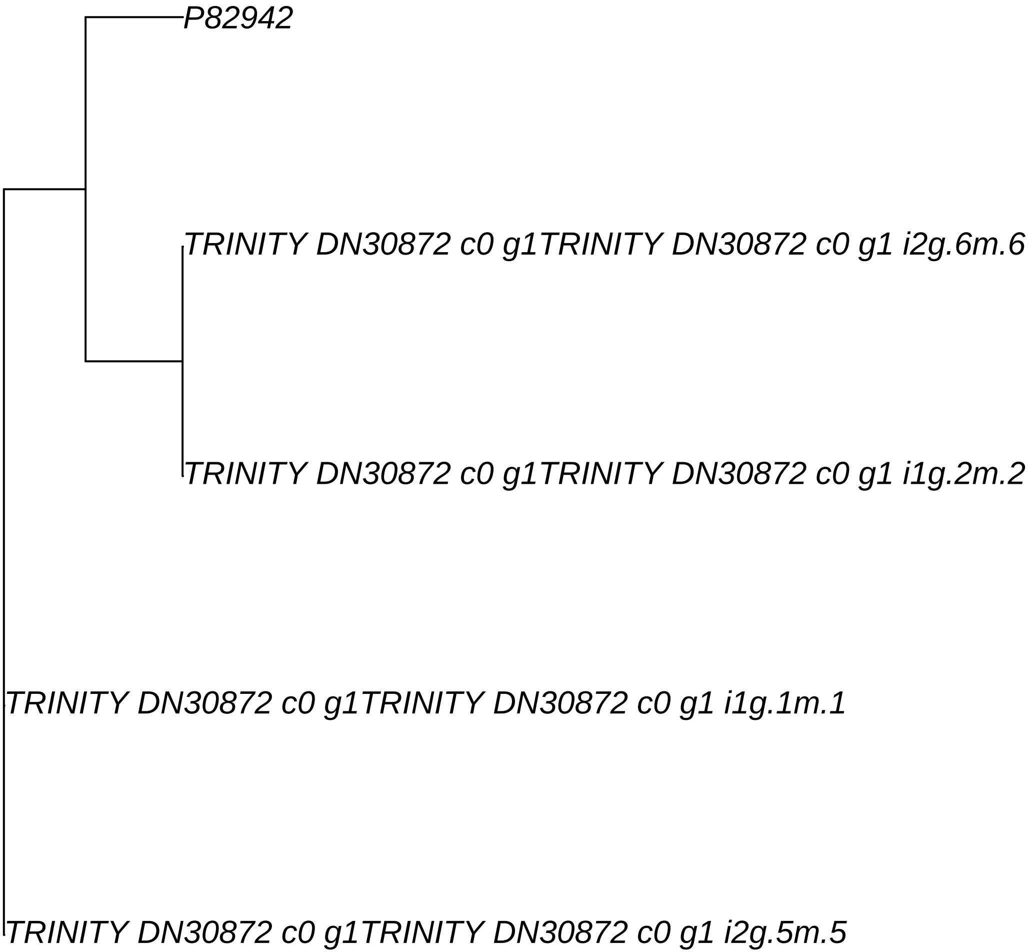
P82942
TRINITY DN30872 c0 g1TRINITY DN30872 c0 g1 i2g.6m.6
TRINITY DN30872 c0 g1TRINITY DN30872 c0 g1 i1g.2m.2
TRINITY DN30872 c0 g1TRINITY DN30872 c0 g1 i1g.1m.1
TRINITY DN30872 c0 g1TRINITY DN30872 c0 g1 i2g.5m.5
